# Meta-Transcriptomic Analysis of RNAseq Data Reveals Pacu and Loach Fish with Unusually High Levels of Myoglobin Expression in Skeletal Muscles

**DOI:** 10.3390/ani10071130

**Published:** 2020-07-03

**Authors:** Rui-Yi Chen, Bui Thi Ngoc Hieu, Gilbert Audira, Bao Lou, Ming-Der Lin, Chung-Der Hsiao

**Affiliations:** 1 Key Lab of Mariculture and Enhancement of Zhejiang Province, Marine Fisheries Research Institute of Zhejiang, Zhoushan 316100, China; xx_cry@163.com; 2 Marine and Fishery Institute, Zhejiang Ocean University, Zhoushan 316100, China; 3 Department of Bioscience Technology, Chung Yuan Christian University, Chung-Li 32023, Taiwan; hieubtn90@gmail.com (B.T.N.H.); gilbertaudira@yahoo.com (G.A.); 4 Department of Chemistry, Chung Yuan Christian University, Chung-Li 32023, Taiwan; 5 Institute of Hydrobiology, Zhejiang Academy of Agricultural Sciences, Shiqiao Road 198, Hangzhou 310021, China; 6 Department of Molecular Biology and Human Genetics, College of Medicine, Tzu Chi University, 701 Zhongyang Rd, Sec. 3, Hualien 97004, Taiwan; 7 Center for Nanotechnology, Chung Yuan Christian University, Chung-Li 32023, Taiwan

**Keywords:** myoglobin, hemoglobin, fish, meta-transcriptomic

## Abstract

**Simple Summary:**

Oxygen-binding proteins that mediate oxygen-binding for storage and consumption, to reduce energy, are very diverse in fish, depending on their habitats. In the present study, oxygen-binding protein gene expression in the skeletal muscle of 25 diverse fish species was examined by a meta-transcriptomic approach. By using RNAseq data, this is the first study to examine the high level of myoglobin, one of the oxygen-binding proteins, transcripts in pacu and loach fish that might be related to their high tolerance for the oxygen-deficient environment. In addition, this study presents the power of the current method to compare the fish oxygen-binding protein expression and its putative gene expansion event.

**Abstract:**

Oxygen-binding proteins, such as myoglobin, hemoglobin, neuroglobin, and cytoglobin, play a role in oxygen binding and delivery to tissues. In icefish, the loss of myoglobin and hemoglobin genes has been reported to be an adaptive evolution event. This interesting finding prompted us to exam oxygen-binding protein expression in diverse fish species. Taking advantage of substantial RNAseq data deposited in the NCBI (National Center for Biotechnology Information) database, we adopted a meta-transcriptomic approach to explore and compare four oxygen-binding protein gene expression levels in the skeletal muscle of 25 diverse fish species for the first time. RNAseq data were downloaded from the NCBI Sequence Read Archive (SRA) database, and de novo assembly was performed to generate transcript contigs. The genes encoding oxygen-binding proteins were then identified by the BLAST search, and the relative expression level of oxygen-binding protein genes was normalized by the RPKM (Reads per Kilobase Million) method. By performing expression profiling, hierarchy clustering, and principal component analysis, pacu and loach fish were noticed by their high myoglobin expression levels in skeletal muscle tissues among 25 diverse fish species. In conclusion, we demonstrated that meta-transcriptomic analysis of RNAseq data is an informative approach to compare the oxygen-binding protein expression and putative gene expansion event in fish.

## 1. Introduction

Oxygen homeostasis is an essential mechanism in living organisms that is regulated by four major oxygen-binding proteins, namely hemoglobin, myoglobin, cytoglobin, and neuroglobin [1]. The primary classification of these four oxygen-binding proteins is based on their tissue distribution and oxygen-binding affinity. The major role of these oxygen-binding proteins is on mediating oxygen-binding for storage and consumption, to reduce energy by respiratory progress. Hemoglobin is a tetramer comprising two α and two β globin chains (α2β2), one molecule of heme, and ferric ions. Hemoglobin is essential for aerobic-respiratory fishes, whose living habitats are highly abundant in oxygen [2,3,4]. Myoglobin is a monomer and predominantly expressed in skeletal and cardiac muscles, and it is an essential intermediary factor to mediate an oxygen delivery from blood to muscle tissues [5,6]. Cytoglobin is a hemoprotein expressed in response to oxidative stress in a variety of tissues. It facilitates the diffusion of oxygen through tissues and helps intracellular oxygen transport [7,8]. The neuroglobin is a monomeric globin with a higher oxygen affinity than hemoglobin. It has been reported that neuroglobin plays a protective role against nitric oxide toxicity in the nervous system [9,10].

Fish are the most widespread and divergent vertebrates that adopted to diverse habitats with different salinity (freshwater, brackish water, or seawater), distinct dissolved oxygen, alkalinity, and temperatures. Some freshwater species, such as crucian carp (*Carassius carassius*) can survive in extremely oxygen-deficient conditions by relying more on subtle neuromodulatory mechanisms, such as ethanol production as their metabolic end-product, caused by duplicated pyruvate dehydrogenase genes, for anoxic metabolic depression [11,12]. In addition, another freshwater species, the pacu fish (*Piaractus mesopotamicus*), which has a high economic value in South America, utilizes aquatic surface respiration (ASR) in order to survive in this condition [13]. Moreover, this species can also survive in conditions with less than 9 mm Hg oxygen concentrations under the intensive aquaculture conditions in fishponds. There is increasing attention being given to the pacu fish, particularly from researchers from Brazil; this interest enhances the farming of this species. Accordingly, some studies on the reproduction [14], feeding, and nutrition [15,16,17] of pacu fish have been carried out [18]. Several fish species have been evolved in dealing with hypoxia or even anoxia conditions, such as pond loach (*Misgurnus anguillicaudatus*), by aquatic surface respiration [19], and Siamese fighting fish (*Betta splendens*), by labyrinth organ respiration [20]. Instead of the tolerance of hypoxia conditions, the Atlantic cod (*Gadus morhua* L.) displays avoidance behavior from the hypoxia environment, by enhancing oxygen sensing [21].

Gene expression profiling based on the difference in expression levels, such as microarray, which detects the upregulation or downregulation of target genes in certain physiological or pathological conditions in fish, is widely used in the study of biological processes [22,23,24]. However, one major disadvantage of such a microarray-based transcript detection method lies in its limitation on detecting transcripts with low expression levels. In addition, due to the limitation of the probe density in a single chip, the gene of interest could be missed if the corresponding probe does not exist on the chip. Compared to the microarray-based method, RNAseq deep sequencing provides a complete transcript content and thus can quantify gene expression according to a given reference genome. Therefore, the next-generation sequencing techniques for RNAseq have become an alternative or even better tool to the researches in the field of transcriptomic analysis due to its high-throughput, unbiased, and relatively cost-effective nature. Taken together, by taking advantage of the abundant RNAseq entries in the NCBI (National Center for Biotechnology Information) Sequence Read Archive (SRA) database, we applied a meta-transcriptomics approach to explore the expression profiling of four oxygen-binding protein genes in skeletal muscle from 25 diverse fish species.

## 2. Materials and Methods

### 2.1. RNA Extraction

The muscle tissues dissected from wild juvenile Bombay duck (body length around 2 cm) and beltfish (*Trichiurus lepturus*) were immediately stored in RNAlater (Qiagen, Hilden, Germany) and then stored at −80 °C, prior to RNA extraction. Total RNAs were extracted by using the TRIZOL Kit (Invitrogen, Carlsbad, CA, USA), following the manufacturer’s instructions. Total RNA samples were then digested by DNase I (Thermo Scientific, Waltham, MA, USA), to remove potential genomic DNA contamination. Integrity and size distribution were checked with a Bioanalyzer 2100 (Agilent Technologies, Santa Clara, CA, USA) (with ethics document approval ID CYCU104024).

### 2.2. RNA Isolation, Library Construction, and Illumina Sequencing

Initially, about 2.5 µg of starting total RNAs was used to synthesize the cDNA libraries, by following the standard protocols of the Illumina TruSeq RNA Sample Preparation Kit (Illumina, San Diego, CA, USA). The final library had an insert size of about 200–300 bp. After qPCR quantitation and dilution (by using KAPA SYBR FAST Master Mix), the sequencing library was sequenced on an Illumina NextSeq500 platform with 150 bp paired-end reads. Adaptor sequences were trimmed, and reads with low quality were further removed by cutadapt software [25]. After the removal of ambiguous nucleotides, duplicates, and low-quality sequences (Phred quality scores < 20), cleaned reads were obtained and deposited in the NCBI SRA database (Table 1).

### 2.3. De Novo Assembly of RNAseq

Other fish muscles’ raw next-generation sequencing (NGS) reads were downloaded from the file transfer protocol (FTP) site of Sequence Read Archive (SRA) in the DNA Data Bank of Japan (DDBJ) database (http://trace.ddbj.nig.ac.jp/dra/index_e.html) by FileZilla software (https://filezilla-project.org/) and then converted into fastq format by using NCBI sratoolkit (http://www.ncbi.nlm.nih.gov/Traces/sra/?view=software). The raw NGS reads of muscle from each fish species were de novo assembled into contigs by CLCBio software (http://www.clcbio.com/) with default parameter settings (k-mer = 23 and bubble size = 50). The number and quality (N50) of assembled contigs for all analyzed fish species are summarized in Table 1.

### 2.4. Identification of Oxygen-Binding Proteins in Muscle Transcriptome

The assembled contigs were later exported from CLCBio software and imported into Geneious software, to perform in-house basic local alignment search tool (BLAST), to find regions of local similarity between sequences. We used tBLASTN, a mode operation for BLAST that aligns protein sequences to a nucleotide database translated in all six frames, to search the potential contigs coding for either myoglobin, hemoglobin, cytoglobin, or neuroglobin with an e-value cutoff setting at 1 × 10^−5^. The potential contig was later BLASTX against the reference sequence (Refseq) database, a comprehensive, integrated, non-redundant, well-annotated set of reference sequences, including genomic, transcript, and protein, to validate the gene annotation with an e-value cutoff of 1 × 10^−5^. For gene expression calculation, the raw reads were mapped to the assembled contigs by CLCBio software, and the relative expressional level was calculated as RPKM (Reads per Kilobase Million). The number and quality (N50) of the oxygen-binding-related proteins of 25 analyzed fish species were summarized in Table 1.

### 2.5. PCA, Heatmap, and Clustering Analysis

In order to compare the relative gene expression patterns of four oxygen-binding proteins in muscle tissues among 25 different fish species, PCA was performed, and heatmap clusters were generated. The relative contig number and RPKM values for each oxygen-binding protein were input into and saved as an Excel file (.xlsx), by using Microsoft Excel, and the Excel file was converted to a comma-separated values-type file (.csv) to be readable by R software. PCA, heatmap, and clustering analysis were carried out by R software (https://www.r-project.org/). In addition, mean centering was carried out, since each variable was treated equally, in the sense that larger relative changes in response will receive the most weighting. In the PCA, variable group classification was determined by using the k-means clustering algorithm, and then the clusters returned by the k-means algorithm were used to color variables [26].

## 3. Results and Discussion

### 3.1. Meta-Transcriptomic Analysis of Oxygen-Binding Protein Expression

**Figure 1:**
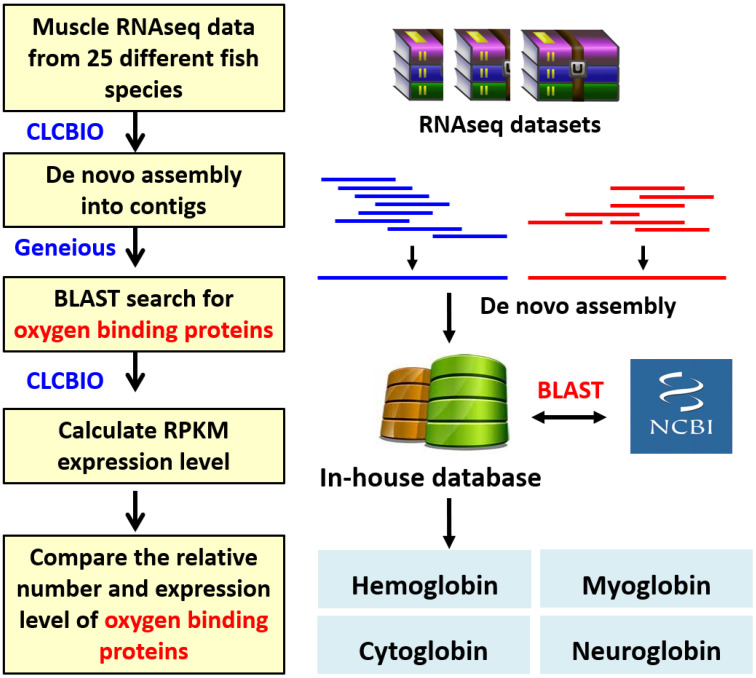
Summary of the experimental workflow from bioinformatic analysis of the expression of oxygen-binding proteins in fish. We performed de novo gene assembly, gene mining by BLAST search, and gene mapping to compare the expressional level of four major oxygen-binding proteins (hemoglobin, myoglobin, cytoglobin, and neuroglobin) in 25 different fish species.

Fish can survive in a diverse range of water oxygen levels. By radiation adaptation, the expression of hemoglobin and myoglobin genes is absent in some Antarctic icefishes [29,30]. This interesting phenomenon led us to ask whether the expression levels of oxygen-binding protein genes display a unique pattern among various fish species that live in a diverse habitat with different water oxygen levels. To answer this question, we used a bioinformatics approach to compare the relative expression levels of two major oxygen-binding protein genes, *hemoglobin* and *myoglobin*, in the skeletal muscle of fish. In addition, other two oxygen-binding protein genes, *cytoglobin* and *neuroglobin* were also analyzed, in order to serve as control groups. Taking the advantage of the rich information in the Sequence Read Archive (SRA) database, we sequenced or collected muscle RNAseq data from 25 fish species, to perform comparative studies systematically (species names are listed in Table 1). We used CLCBio software to assemble raw RNAseq reads into contigs by using the default setting (analysis pipeline is summarized in Figure 1). The detailed information for fish species, raw data size, sequencing method, assembly contig number, and assembly quality (N50) were summarized in Table 1. The size of raw SRA data for different fish species ranged from 1.9 to 8.1 Gb. The size of the assembled contig number for different fish species ranged from 21,259 to 136,066, with N50 ranging from 338 to 972. The oxygen-binding protein homologs of hemoglobin, myoglobin, cytoglobin, and neuroglobin were identified by using BLAST search, and the relative number of assembled contigs matched with oxygen-binding proteins for different fish species is summarized in Appendix A, Table A1. In RNAseq datasets of fish skeletal muscle, based on relative gene expression level, we found the predominant oxygen-binding proteins expressed are hemoglobin and myoglobin. On the contrary, the number of annotated contigs encoding cytoglobin or neuroglobin is very low or even absent in most fish species (Appendix A, Table A1). Among 25 fish species analyzed, we found the pacu fish (*Piaractus mesopotamicus*) displays a unique oxygen-binding protein expression due to its outstanding high number for assembled contigs annotated as either *hemoglobin* (65 transcripts) and *myoglobin* (31 transcripts) (Appendix A, Table A1). By contrast, the contig numbers of *hemoglobin* and *myoglobin* in other fish species are usually less than 16 and 6, respectively (Appendix A, Table A1).

### 3.2. Comparison of the Expression Levels of Oxygen-Binding Protein Genes among Fish Species

**Figure 2:**
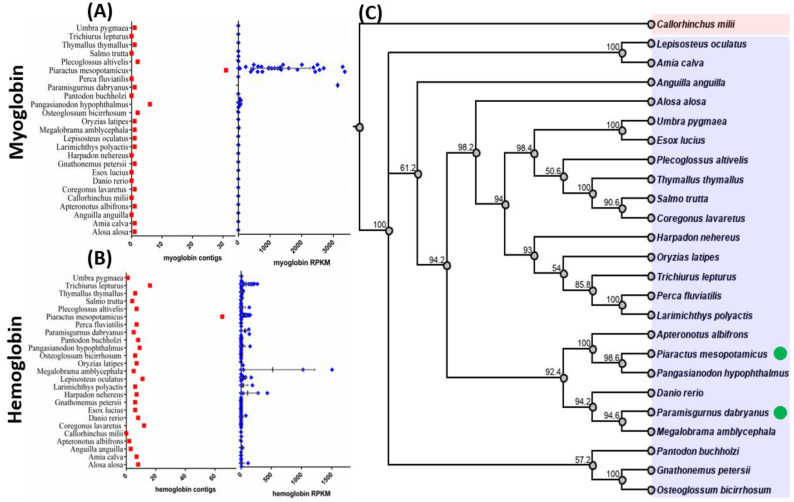
The assembled contig number and relative expression levels of myoglobin and hemoglobin of 25 diverse fish species. (**A**) The gene contig number (red color) and expression level (blue color) comparison for myoglobin in fish. (**B**) The gene contig number (red color) and expression level (blue color) comparison for hemoglobin in fish. The gene expression level was normalized and compared by Reads per Kilobase Million (RPKM) values. (**C**) The phylogenetic tree based on complete mitochondrial DNA sequences from 25 fish species. The multiple sequence alignment and phylogenetic tree construction were performed by Geneious software and bootstrap value, to support the tree topology, and are indicated in the root position of each branch. The red- and blue-shaded color column represents cartilaginous and teleost lineages, respectively. The green dots represent two fish species that were identified with high myoglobin expression in the skeletal muscle.

Next, we performed gene mapping to compare the relative expression level of *hemoglobin* and *myoglobin* in skeletal muscle tissues among diverse fish species. The relative expression level of oxygen-binding protein homologs was calculated and presented as RPKM (Reads per Kilobase Million) for expressional normalization [31]. As a result, we identified that the pacu fish has a high *myoglobin* expression level of 1327.8, based on RPKM (Figure 2A, Appendix A, Table A2). Although the number of contigs annotated as *hemoglobin* is high in pacu fish, their relative expression level is low (Figure 2B). Another interesting species is the loach fish (*Paramisgurnus dabryanus*), showing one *myoglobin*-associated contig, but with an extremely high RPKM expression level, at 3136.4 (Figure 2A, Appendix A, Table A2). Based on hemoglobin transcript abundance, we found the Blunt snout bream (*Megalobrama amblycephala*), an oxygen-sensitive fish species, displays a relatively high hemoglobin expression level in the skeletal muscle (Figure 2B). The other two oxygen-binding protein genes, *cytoglobin* and *neuroglobin* can be considered as low-expressed genes in skeletal muscle since their RPKM level is less than 2.5 in all fish species examined. The phylogenetic tree based on complete mitochondrial DNA sequences from these fish species can be found in Figure 2C.

### 3.3. PCA and Hierarchical Clustering Analyses of the Oxygen-Binding Protein Genes Expression among Fish Species

**Figure 3:**
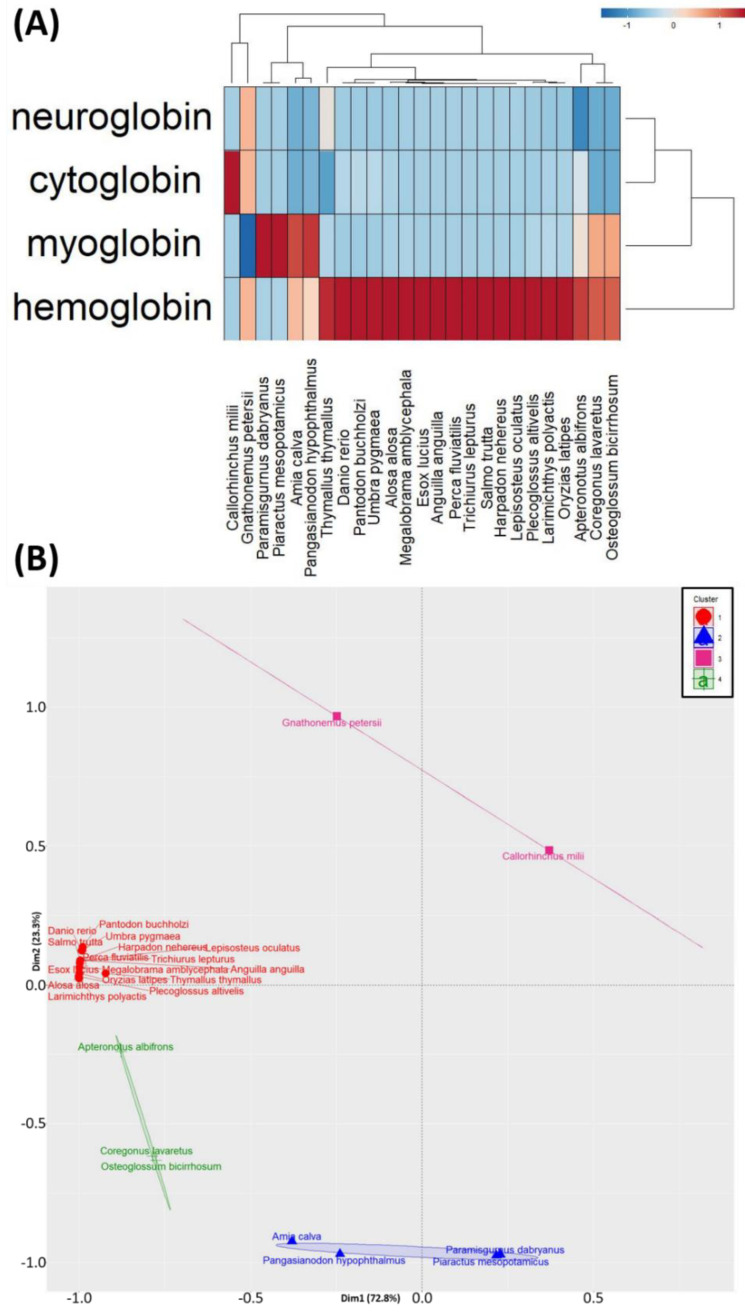
Comparison of the relative oxygen-binding protein gene expression among 25 diverse fish species. (**A**) Hierarchical clustering analysis of the relative oxygen-binding protein gene expression levels among 25 diverse fish species. (**B**) Principal component analysis (PCA) of the relative oxygen-binding protein gene expression levels among 25 diverse fish species. Totally, 25 fish species can be categorized into four major groups (shown by different color labeling). Pacu (*Piaractus mesopotamicus*) and loach (*Paramisgurnus dabryanus*) fish were identified with high myoglobin expression in skeletal muscle and were grouped in the second group (blue color).

Next, we performed principal component analysis (PCA) and hierarchical clustering, to reduce the data dimension and complexity, and to generate a heatmap for inter-species comparison. PCA is a mathematical method able to reduce data dimension and complexity. From the PCA analysis, all of the 25 fishes were grouped into four major clusters, based on their gene expression patterns for *hemoglobin*, *myoglobin*, *cytoglobin*, and *neuroglobin*, with the first cluster (red color) as the biggest cluster among all the clusters (Figure 3B). The majority of the fishes, including zebrafish and medaka fish, were grouped in the first cluster (red color) (Figure 3B), which has higher *hemoglobin* expression than the fish species in other clusters (Figure 3A). Four fishes, namely *Amia calva*, *Pangasianodon hypophtaimus*, *Parmisgurpus dabryanus*, and *Piaractus mesopotamicus*, were grouped into the second cluster (blue color). Based on the heatmap results, it can be concluded that this grouping is reasonable because these fishes have unique and similar patterns regarding their *myoglobin* expression level, which is quite high compared to the rest of the fishes (Figure 3A). The third cluster (pink color) consists of two species, *Gnathonemus petersii* and *Callorhinchus milii*. This categorization is plausible since both of the fishes showed a high level of *cytoglobin* expression in the skeletal muscle, while this phenomenon was not observed in other fishes (Figure 3A). Furthermore, the fourth cluster (green color) consists of *Apteronotus albifrons*, *Coregonus lavaretus*, and *Osteglossum bicirrhosum*, showing a high-level expression of both *myoglobin* and *hemoglobin* in their skeletal muscle (Figure 3A). Taken together, the different expression levels of oxygen-binding protein genes in each fish species examined resulted in four major clusters in the PCA and heatmap clustering analyses.

### 3.4. The Skeletal Muscle of Pacu Fish Has Multiple Myoglobin Transcripts with High Expression Level

**Figure A1:**
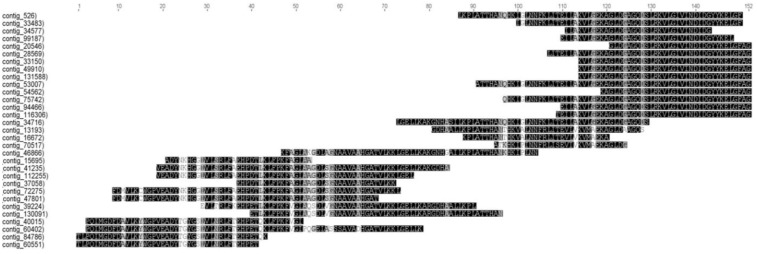
Sequence alignment of the translated contigs annotated as myoglobin in pacu fish. Thirty-one polypeptide translated from *myoglobin* contigs were used for multiple sequence alignment by using Geneious software. The conserved residues are marked as shaded black color, and the non-conserved residues are marked as open or gray-shaded colors.

Our meta-transcriptomic study provides molecular evidence that there is a high level of *myoglobin* expression in the skeletal muscle of pacu fish. This finding may help to explain their ability to cope with oxygen-deficiency habitats. Moreover, we identified 31 transcripts annotated as *myoglobin*. The numerous forms of *myoglobin* transcripts identified in the pacu fish muscle could be originated by a large number of duplications of *myoglobin* genes in its genome. In common carp, duplication and differentiation of *myoglobin* genes have been identified by bacterial artificial chromosome (BAC) analysis [32]. In West African lungfish, *Protopterus annectens*, at least seven distinct *myoglobin* genes (PanMb1–7) have been identified, and each of them displays differential expression patterns in different tissues [33]. Consistent with the hypothesis that *myoglobin* genes are highly duplicated in the pacu genome, we observed several non-redundant *myoglobin* transcripts encoding myoglobin with amino acid substitutions by multiple sequence alignment (Appendix A, Figure A1). Alternatively, we hypothesize that multiple *myoglobin* transcript contigs in pacu fish may be generated by alternative splicing. This hypothesis is based on a previous study that detected sixteen alternatively-spliced *myoglobin* transcripts in human cancer [34]. Future studies on whole-genome sequencing for pacu fish are required to validate the above hypotheses. In addition to *myoglobin*, this study also identified relative high-level *hemoglobin* expression in the skeletal muscle of blunt snout bream (*Megalobrama amblycephala*). Blunt snout bream is an oxygen-sensitive fish species showing massive mortality once the dissolved oxygen level declines in the pond. Therefore, the high-level hemoglobin expression in the skeletal muscle in blunt snout bream can be considered to be a physiological adaptation. More studies are needed to address the underlying mechanism for this interesting phenotype.

## 4. Conclusions

In conclusion, in this study, we provided good evidence to show the power of meta-transcriptomic analysis on mining and comparing oxygen-binding protein expression in fish muscle RNAseq data for the first time. This approach led us to identify pacu and loach fish with exceptional levels of *myoglobin* transcripts in their skeletal muscle that might be functionally associated with their high-tolerance potential for the oxygen-deficient environments. However, one has to keep in mind that deposited datasets in the SRA that were used in the present study often represent a relatively small number of biological samples, and there is a possibility that the different animals included were maintained under identical or comparable conditions. Thus, validation with RT-qPCR for the fish species is needed in the future. In addition, this approach opens up a new avenue to perform data mining on more species or tissue types, to validate *myoglobin* and *hemoglobin* expressional adaptation among diverse fish species.

## Figures and Tables

**Table 1:** Summary of the fish species and muscle transcriptomic data used in this study.

SRR ID	Species Name	Data Size (Gb)	Sequencing Platform	Sequencing Method	Assembled Contigs	N50
SRR1613325	*Megalobrama amblycephala*	2.3	Illumina HiSeq 2000	2X101 bp	35052	557
SRR1652342	*Paramisgurnus dabryanus*	3.6	Illumina HiSeq 2000	2X102 bp	49125	972
SRR1533666	*Thymallus thymallus*	3	Illumina HiSeq 2000	2X101 bp	102377	489
SRR1533644	*Pangasianodon hypophthalmus*	3.6	Illumina HiSeq 2000	2X101 bp	86400	454
SRR1056356	*Piaractus mesopotamicus*	8.1	Illumina HiSeq 2000	2X101 bp	131747	483
SRR514104	*Callorhinchus milii*	6.4	Illumina Genome Analyzer II	2X77 bp	44417	338
SRR1524253	*Lepisosteus oculatus*	3.7	Illumina HiSeq 2000	2X101 bp	82408	517
SRR1524264	*Amia calva*	4.1	Illumina HiSeq 2000	2X101 bp	94702	554
SRR1524274	*Oryzias latipes*	4.6	Illumina HiSeq 2000	2X101 bp	100420	560
SRR1532750	*Apteronotus albifrons*	3.6	Illumina HiSeq 2000	2X101 bp	124794	551
SRR1532760	*Anguilla anguilla*	2.7	Illumina HiSeq 2000	2X101 bp	86397	429
SRR1532781	*Salmo trutta*	5.5	Illumina HiSeq 2000	2X101 bp	121092	486
SRR1532792	*Osteoglossum bicirrhosum*	3.2	Illumina HiSeq 2000	2X101 bp	107973	469
SRR1532803	*Alosa alosa*	4.8	Illumina HiSeq 2000	2X101 bp	115049	502
SRR1532771	*Pantodon buchholzi*	2.6	Illumina HiSeq 2000	2X101 bp	93563	567
SRR1533633	*Umbra pygmaea*	3.2	Illumina HiSeq 2000	2X101 bp	107513	583
SRR1533655	*Esox lucius*	4	Illumina HiSeq 2000	2X101 bp	73132	550
SRR1533677	*Coregonus lavaretus*	3.9	Illumina HiSeq 2000	2X101 bp	93899	481
SRR1533689	*Perca fluviatilis*	5.1	Illumina HiSeq 2000	2X101 bp	75135	563
SRR1533701	*Gnathonemus petersii*	4.3	Illumina HiSeq 2000	2X101 bp	136066	569
SRR1533711	*Plecoglossus altivelis*	3.1	Illumina HiSeq 2000	2X101 bp	115114	537
SRR3029231	*Larimichthys polyactis*	1.9	Illumina HiSeq 2000	2X101 bp	60237	715
SRR1524241	*Danio rerio*	4.3	Illumina HiSeq 2000	2X101 bp	118459	509
SRR332188^ 1^	*Trichiurus lepturus*	5.7	Illumina NextSeq 500	2X151 bp	36214	860
SRR338795^ 1^	*Harpadon nehereus*	2.3	Illumina NextSeq 500	2X151 bp	21599	615

^1^ Those two RNAseq data were performed in the current study and were partly reported in our previous publications [27,28]. The other 23 RNAseq data were downloaded from the public Sequence Read Archive (SRA) database.

## Appendix A

**Table A1:** Summary of the assembled contigs annotated as transcripts of oxygen-binding protein genes in 25 fish species.

Species Name	Hemoglobin	Myoglobin	Cytoglobin	Neuroglobin
*Alosa alosa*	8	1	2	0
*Amia calva*	7	1	0	0
*Anguilla anguilla*	3	0	0	0
*Apteronotus albifrons*	2	1	1	0
*Callorhinchus milii*	0	0	1	0
*Coregonus lavaretus*	12	1	0	0
*Danio rerio*	8	0	2	0
*Esox lucius*	6	0	0	0
*Gnathonemus petersii*	6	1	1	1
*Harpadon nehereus*	7	0	0	0
*Larimichthys polyactis*	6	1	1	0
*Lepisosteus oculatus*	11	1	0	0
*Megalobrama amblycephala*	5	1	0	0
*Oryzias latipes*	7	1	2	0
*Osteoglossum bicirrhosum*	6	2	0	0
*Pangasianodon hypophthalmus*	9	6	0	0
*Pantodon buchholzi*	8	0	2	0
*Paramisgurnus dabryanus*	5	1	0	0
*Perca fluviatilis*	7	0	0	0
*Piaractus mesopotamicus*	65	31	0	0
*Plecoglossus altivelis*	7	2	1	0
*Salmo trutta*	4	0	0	0
*Thymallus thymallus*	6	1	2	1
*Trichiurus lepturus*	16	0	0	0
*Umbra pygmaea*	1	1	1	0

**Table A2:** Summary of the transcript expression level based on the RPKM method for oxygen-binding protein genes in 25 fish species.

Species Name	Hemoglobin	Myoglobin	Cytoglobin	Neuroglobin
*Alosa alosa*	16.1	0.6	0.8	0
*Amia calva*	0.5	0.8	0	0
*Anguilla anguilla*	14.7	0	0	0
*Apteronotus albifrons*	2.2	1.1	0.9	0
*Callorhinchus milii*	0	0	2.4	0
*Coregonus lavaretus*	0.4	0.3	0	0
*Danio rerio*	10.4	0	0.7	0
*Esox lucius*	0.4	0	0	0
*Gnathonemus petersii*	0.3	0.2	0.3	0.3
*Harpadon nehereus*	108.5	0	0	0
*Larimichthys polyactis*	38.6	3.9	2.5	0
*Lepisosteus oculatus*	37.2	0	0	0
*Megalobrama amblycephala*	523.3	14.0	0	0
*Oryzias latipes*	5	0.4	0.3	0
*Osteoglossum bicirrhosum*	1.3	1	0	0
*Pangasianodon hypophthalmus*	20.8	43.5	0	0
*Pantodon buchholzi*	2.2	0	0.2	0
*Paramisgurnus dabryanus*	67.6	3136.4	0	0
*Perca fluviatilis*	1.4	0	0	0
*Piaractus mesopotamicus*	46	1327.8	0	0
*Plecoglossus altivelis*	20.8	1.4	0.3	0
*Salmo trutta*	4.5	0	0	0
*Thymallus thymallus*	13.6	3.2	0.3	5.6
*Trichiurus lepturus*	81.9	0	0	0
*Umbra pygmaea*	8.4	0.12	0.8	0
